# Utilisation of priority traditional medicinal plants and local people's knowledge on their conservation status in arid lands of Kenya (Mwingi District)

**DOI:** 10.1186/1746-4269-6-22

**Published:** 2010-08-16

**Authors:** Grace N Njoroge, Isaac M Kaibui, Peter K Njenga, Peter O Odhiambo

**Affiliations:** 1 Jomo Kenyatta University of Agriculture and technology, Botany department, P.O. Box 62000 Nairobi, Kenya

## Abstract

Mwingi District lies within the Kenyan Arid and Semiarid lands (ASALs) in Eastern Province. Although some ethnobotanical surveys have been undertaken in some arid and semiarid areas of Kenya, limited studies have documented priority medicinal plants as well as local people's awareness of conservation needs of these plants. This study sought to establish the priority traditional medicinal plants used for human, livestock healthcare, and those used for protecting stored grains against pest infestation in Mwingi district. Further, the status of knowledge among the local people on the threat and conservation status of important medicinal species was documented. This study identified 18 species which were regarded as priority traditional medicinal plants for human health. In terms of priority, 8 were classified as moderate, 6 high, while 4 were ranked highest priority species. These four species are *Albizia amara *(Roxb.) Boiv. (Mimosacaeae), *Aloe secundiflora *(Engl. (Aloaceae), *Acalypha fruticosa *Forssk. (Euphorbiaceae) and *Salvadora persica *L. (Salvadoraceae).

In regard to medicinal plants used for ethnoveterinary purposes, eleven species were identified while seven species were reported as being important for obtaining natural products or concoctions used for stored grain preservation especially against weevils. The data obtained revealed that there were new records of priority medicinal plants which had not been documented as priority species in the past. Results on conservation status of these plants showed that more than 80% of the respondents were unaware that wild medicinal plants were declining, and, consequently, few of them have any domesticated species. Some of the species that have been conserved on farm or deliberately allowed to persist when wild habitats are converted into agricultural lands include: *Croton megalocarpus *Hutch., *Aloe secundiflora*, *Azadirachta indica *A. Juss., *Warburgia ugandensis *Sprague, *Ricinus communis *L. and *Terminalia brownie *Fresen. A small proportion of the respondents however, were aware of the threats facing medicnal plants. Some of the plants reported as declining include, *Solanum renschii *Vatke (Solanaceae), *Populus ilicifolia *(Engl.) Rouleau (Salicaceae), *Strychnos henningsii *Gilg (Loganiaceae) and *Rumex usambarensis *(Dammer) Dammer (Polygonaceae). Considering the low level of understanding of conservation concerns for these species, there is need therefore, to build capacity among the local communities in this area particularly in regard to sustainable use of natural resources, conservation methods as well as domestication processes.

## Introduction

In promotion of conservation agenda, it is important to understand how local communities use and manage natural resources. Studies in ethnobiology (including Ethnobotany) and traditional ecological knowledge are known to serve as significant bridges between conservation scientists and local communities. These studies help in understanding how local communities relate to their environment and hence, pave a way for their active involvement in natural resource conservation [1]. Empowerment of local communities to conserve and sustainably use biodiversity is increasingly becoming an important policy shift as most of the local people in rural areas depend on natural resources for their livelihoods [2].

The use and commercialisation of traditional medicinal plants, has been found to be an important livelihood strategy, in developing countries where rural people are economically vulnerable [3], hence improving incomes and living standards [4]. In the trade with *Prunus africana*, for example, significant improvement of village revenues has been reported [5]. Harvesting of wild resources is therefore an economic activity recognised both locally and internationally, leading to revision of planning initiatives with the view to provide enabling economic policy frameworks [6]. Drylands have some of the highest poverty levels, for example northern part of Kenya, has an incidence of poverty as high as 84% [7]. Wild habitats especially, forests are known to provide support to about 1.4 billion people globally who live on USD$ 2 or less per day [8] Policy changes now recognise local communities as principal actors in biodiversity use and conservation. It is hoped that this will strike a balance between satisfying the livelihood needs and wise use of natural resources to ensure sustainable development. An often unanswered question is the extent to which local people perceive conservation agenda.

The communities who make use of natural resources usually have interacted with different components of biodiversity over the years, and hence accumulated important traditional knowledge regarding their use. Ethnobotanical studies have revealed vital information on how local people utilise plants for various purposes over time. Collecting ethnobotanical data is therefore, an essential component in sustainable natural resource management, particularly, in regard to medicinal plants usage which provides a large amount of traditional medicines.

Traditional medicines form a central component in health care systems in developing countries where 80% of the population has been reported to depend on traditional medical systems [9]. The use of herbal medicines however, is on the increase even in developed countries because of the belief that herbal remedies are safe because of their natural origin [10]. Globally, there are about 120 plant-derived drugs in professional use; three quarters being obtained from traditional medicinal plants [11]. In Kenya, 90% of the population has used medicinal plants at least once for various health conditions [12]. In other regions such as Peru, it has been found that about 84% of the local people prefer traditional medicinal plants for their health care needs in comparison to modern pharmaceutical products. Some of the reasons given include the fact that they are of natural origin and no risks or harm is experienced when used [13].

Unfortunately, according to a recent report, almost one third of medicinal plant species could become extinct, with losses reported in China, India, Kenya, Nepal, Tanzania and Uganda [14]. Greater losses are expected to occur in arid and semi arid areas due to factors such as: climate change, erosion, expansion of agricultural land, wood consumption, and exploitation of natural vegetation, increased global trade in natural resources, domestication, selection and grazing among other factors [15,16].

As demand for medicinal plants continue to accelerate, species preservation is perceived to depend on sustainable harvesting methods and cultivation. The importance of *Prunus africana *in pharmaceutical industry in the west and its consequent depletion in the wild has caused it to be popularised as a "cash crop" in African countries particularly Cameroon and Kenya [17]. In the Peruvian Amazon, important medicinal plants especially those in commercial exploitation are currently being obtained from cultivation. It is therefore being realised that cultivation of medicinal plant species may be the only solution for their rapid conservation [18]. In Asia, more and more medicinal plant species are being depleted, some becoming endangered; hence cultivation is being viewed as a viable alternative source of these resources, despite challenges in ex-situ management strategies [19,20].

Although drylands are sometimes viewed as "wastelands" [21], they contain species of immense scientific, economic and social value and are unique in that they are adapted to survive under extreme environmental conditions [15,22]. Dryland species especially medicinal plants (often referred to as "green pharmacy") and health foods are becoming commercialised in this age of health-consciousness [23] hence exerting pressure on the wild resources where most are obtained.

In Kenya ASALs account for 88% of the land's surface area and are home to over 10 million people [24]. These areas are facing intense degradation due to pressure arising from over harvesting of wild plants to generate income. Mwingi district is one of the areas in Eastern Province lying under the ASALs of Kenya. Although a few ethnobotanical studies have been undertaken in the drylands of Kenya, these have focused on utilisation of wild fruits and their potential for commercialisation [25-28]. Furthermore, limited studies have reported local people's perception of conservation status of medicinal plants. Ethnobotanical studies that have reported medicinal plants usage in Eastern Province have focused on Embu, Mbeere, Makueni and Machakos districts, leaving out Mwingi district which is more interior and likely to have more intact traditional knowledge. A recent study in Mbooni forest, Makueni district, indicated that nearly all households in the area harvest Non-Wood Forest Products (NWFPs), half of which are medicines but only small amounts are harvested from farmlands [29].

The documentation of medicinal plants prioritised by the local people, as well as their understanding of possible biodiversity loss and strategies of conservation are some of the under-explored aspects in ethnobotanical studies [19]. Further, the extent to which important medicinal are cultivated is often unclear, even in regions where large amounts of medicinal plants are being commercialised [30]. This study explored the prioritised traditional medicinal plants in Mwingi district as well as plants from which concoctions for protecting stored grains against pests are obtained. Loss of grains to pests is a major challenge in drylands of Kenya which is already food insecure arising from erratic rainfall experienced in the region. In addition, the study sought to establish the level of understanding among local communities in the area regarding species loss/decline as well as the extent of medicinal plants on-farm conservation.

## Materials and methods

### Study area

**Figure 1:**
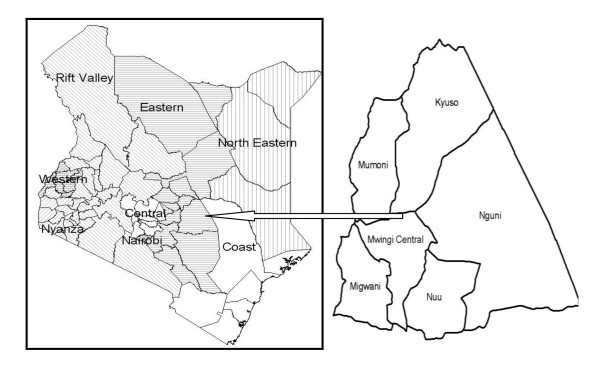
**Mwingi district and constituent divisions where field work was undertaken**.

Mwingi district lies in the arid and semi-arid region of Eastern Province of Kenya and comprises ecologically fragile ecosystems, hence biodiversity assessment and conservation in this region is a priority (Figure 1). The area is inhabited mainly by Kamba people whose vernacular language is referred to as Kikamba. The tribe occupies a large part of Eastern Province and belongs to the Bantu group, with an estimated population of 3 million. Traditionally, the Kamba people were semi-nomadic, and possessed large herds of cattle often practicing limited cultivation. Agriculture however, has now taken over as the primary means of subsistence particularly in the hills where higher amounts of rainfall occurs. River canals have recently been developed in some areas hence facilitating the growing of cereal crops such as sorghum and millet. In the drier areas however, most Kamba people still keep cattle as a means of livelihood. The Kambas are well known for their knowledge of medicinal plants and could be one of the groups in Kenya that has best preserved their traditional knowledge on the use of local plants for medicinal purposes. According to the Mwingi district development plan (2002-2008) and Kenya census of 1999, the district covers an area of 10,030.30 km^2 ^and had an estimated population of 303,828 people. The climate in the district is hot and dry for the greater part of the year, with maximum mean annual temperature ranging from 260 C to 34°C, while minimum mean annual temperatures vary between 14°C and 22°C. Although the rainfall is erratic, it ranges between 400 mm and 800 mm per year. The district shows a very high prevalence of poverty especially, in the drier areas, currently estimated at 60 per cent.

### Data collection

Before embarking on collection of data, on priority medicinal plants in Mwingi district, local community leaders were approached so as to help identify people who were knowledgeable on what the community considers as important medicinal plants. Purposeful sampling technique was used as a tool for identifying key informants familiar with the area and use of natural resources. These comprised individuals who were recommended by community leaders as knowledgeable in the use of traditional medicinal plants and hence were incorporated in the study as field guides. Key informants have been used in earlier studies as guides because they are known to be observant and reflective members of the community who know about the culture and are willing to share their knowledge [31,32].

After this, a younger person was identified as a research assistant. The assistant had to be well known to the people and familiar with the local language. She/he then was taken through the questionnaires, explained the aims of the study before being trusted to conduct the interviews, when the authors were unavailable. The field work was undertaken from January to September, 2006.

After obtaining oral prior-informed consent, in-depth interviews using semi structured questionnaires were administered. In each of the interviews, the respondents were asked to provide a list of five priority medicinal plants species. This was followed by, each respondent being asked to indicate which of the five species they considered most important. In total about 111 respondents were interviewed. The respondents comprised herbalists well known in the area, local resource users who treat their families and friends or use the plants for self-medication. Market visits were made in all the divisions of Mwingi district during the weekly official market days so as to conduct interviews with people selling herbal products. Respondents in the markets as well as those in herbal clinics were randomly sampled by making visits to their premises. Interviews were conducted as long as they agreed to participate. In the rural areas, respondents were identified in consultation with key informants of the specific area. This data was supplemented by participating in systematic natural resource walks and participant observations [33,34].

While in the field, key informants accompanied the researchers to help in identifying and collecting specimens for botanical identification as well as preparation of voucher specimens. In the laboratory identification of the plants was done using the relevant taxonomic literature especially, the family fascles of the Flora of Tropical East Africa (FTEA). Voucher specimens were deposited at the Jomo Kenyatta University herbarium as a reference collection [Additional file 1: Appendix].

The aim of this study was to gather information regarding medicinal plants considered by the community as priority species in human and animal health care well as those used in preservation of stored grains against pests. After listing, the plant species were ranked based on frequency of being mentioned as most important. The number of times each species was cited as most important amongst the listed five species served as our priority index. Species cited between 3-4 times were assigned moderate priority; 5-6 times, high priority and 7 or more times were highest priority criteria. Only plants cited as most important for three or more times were considered in the ranking. Data was also collected to show the level of understanding by the local people regarding threatened or decreasing plant species and any steps they have taken towards conservation.

## Results and Discussion

**Figure 2:**
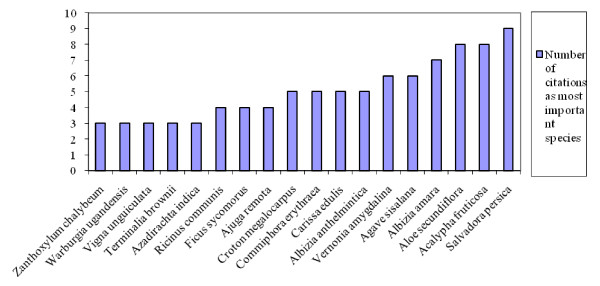
**Number of times medicinal plant species are ranked as most important in Mwingi district, Kenya (N = 91; 3-4 citations-moderate priority species, 5-6 citations-high priority species, ≥ 7- highest priority species)**.

This study identified 18 species which were recognized as priority medicinal plants for human health care. In terms of priority, 8 species were classified as moderate, 6 high while 4 were highest priority species (Figure 2). These four species are *Albizia amara, Aloe secundiflora, Acalypha fruticosa *and *Salvadora persica*. Although all the 18 species are of conservation concern, priority needs to be given to the four species recognised in the highest priority criteria. This high preference rank is known to be an important index in identifying plants of potentially high conservation concern (19).

Previous studies have revealed that ranking as well as frequency of use are important indices in establishing significant plants to the local communities [19,29,35]. A comparison of current priority medicinal plants of Mwingi and other studies elsewhere in the country shows that the composition of prioritised species in this area is unique. Although some species may be regarded as common in other regions, in this listing they were considered priority species. Of the six species listed in Mbooni (Makueni) as important to the local people, [30] none of them was listed as priority species in Mwingi district. Other studies in the province [12] have documented 25 priority medicinal plant species in three districts: Machakos, Makueni and Kitui. Of those only four species are in the list of 18 species prioritised by Mwingi people, and none of these are found among the four species cited in the highest priority criteria. Further, minimal overlaps are revealed in the current study as compared to other documentations in Kenya regarding priority medicinal plants in other regions [35,36]. Studies of Himalayan medicinal plants have revealed that plants from which most medicinal extracts are obtained are habitat specific [37]. This site specific prioritisation and use of local resources needs to be considered when designing conservation and value addition activities for improved sustainable use of medicinal plants.

Direct observations during systematic natural resource walks and participant observations in the field revealed that some medicinal plant species were highly exploited. Some of these are: *Carissa edulis *(Forssk.) Vahl, whose roots are used for management of various ailments (mainly stomach pains) as well as a neutralizer in most herbal preparations; *Warbugia ugadensis*, on the other hand, had its bark stripped off in most of the stocks. During the field surveys, it was observed that *Albizia amara*, which is one of the highly prioritized medicinal plants in this region, was also being extensively harvested as a source of firewood and charcoal. It is possible that this is one of the medicinal plants whose conservation status needs further assessment for purposes of ensuring sustainable supplies for the various sectors that are making demand on it.

In regard to priority ethnoveterinary medicinal plants, eleven species were considered important (Table 1). Of these, eight species were identified to species level but three could only be referred to by their kikamba names. These three plant species had been cited during the interviews and their local names recorded. However when the key respondents joined the researchers in the field to collect those specific plant specimens for purposes of botanical identification and preparation of voucher specimens, they could not be located in the natural habitats. Similar difficulties have been experienced by earlier workers in this province. A study in Mbooni division of Makueni district, for example, revealed that there were cases where cited local plants could not be found in the wild for botanical identification and hence were reported by their local names [29]. It is not clear yet whether these three species were just rare or part of herbalists' confidentiality. Further work is therefore recommended especially, ecological studies involving isolated hills in the district which were inaccessible during this study. This study has realized new priority species list important for ethnoveterinary purposes compared to other studies in Kenya [38]. This then, emphasizes the need for region by region analysis of important traditional medicinal plants both for human and veterinary purposes.

**Table 1:** Priority plant species mostly used for ethnoveterinary purposes in Mwingi district, Kenya

Species/Voucher number	Family	Ethnoveterinary use and method of administration
*Aloe secundiflora *Engl. (GNN *et al*. mwingi 30)	Aloaceae	Leaves cut and steeped in drinking water to control coccidiosis in chicken
*Sclerocarya birrea *(A. Rich.) Hochst. (GNN *et a.l *Mwingi 39)	Anacardiaceae	Bark and roots boiled and a concoction prepared which is topically applied for tick control
*Boscia coriacea *Pax (GNN *et al*. Mwingi 70)	Capparaceae	Leaves crushed and put in water troughs for management of bile problems in chicken
*Juniperus procera *Endl. (GNN *et al*. Mwingi 87)	Cuppressaceae	Sap expressed and applied on flesh wounds of all livestock
*Ricinus communis *L. (GNN *et al*. Mwingi 11)	Euphorbiaceae	Roots boiled and concoction orally administered in management of constipation especially in cattle and goats
*Antidesma venosum *Tul (GNN *et al*. Mwingi 25)	Euphorbiaceae	Sap expressed from the fleshy stems and topically applied on wounds of livestock
*Acacia seyal *Del. (GNN *et al*. Mwingi 26)	Mimosaceae	Bark and roots boiled and orally administered to manage pneumonia in cattle
*Hymenodictyon parvifolium *Oliv. (GNN *et al*. Mwingi 7)	Rubiaceae)	Sap directly applied on infected eyes of livestock
Kikalia		Bark and roots boiled and sprayed on livestock to control ticks
kyangati-		Bark roasted and a powder prepared for management of diarrhea in cattle. Sap also applied to infected eyes
Mwelengwa-		Sap expressed and directly applied on infected eyes of cattle

This study documented seven species which were reported as priority species for preservation of stored grains.These include: *Ocimum gratissimum *L., *Ocimum basilicum *L. (Lamiaceae), *Capsicum frutescens *L.; *C. annuum *L, (Solanaceae), *Maytenus senegalensis *(Lam.) Exell (Celastraceae) as well as two other commonly cited species in local language (*Nyaika*, *Neengia*). These were not identified to species level as it was not possible to obtain voucher specimens to ascertain their botanical identity. The most popular plants in stored grain preservation were found to be *Ocimum gratissimum *and *Ocimum basilicum*. It was also interesting to note that in this study most respondents use ash collected from the fire place to preserve grains especially, against weevils. All the respondents confirmed that they had used local plants for preservation of stored grains; hence, this is a popular method of preserving grains in this region. Previous studies in other regions have shown that plants used by local communities for grain preservation have extracts which have been found to be efficacious against known grain pests [39]. Further work to test activity of plants reported in this study is recommended.

**Figure 3:**
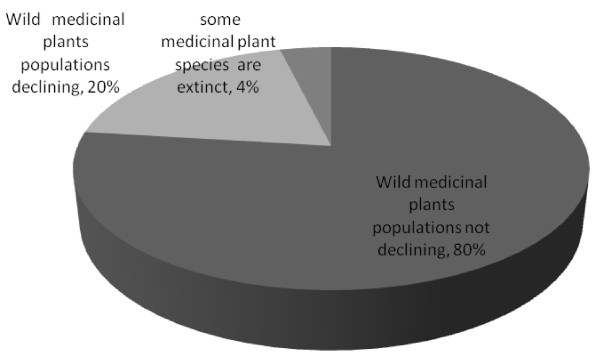
**Knowledge index regarding wild medicinal plants threat among local people at Mwingi district, Kenya (figures refer to percentage of respondents)**.

Results regarding knowledge amongst local people on declining local medicinal plants revealed that more than 80% of the respondents were unaware that wild medicinal plants were declining (Figure 3). During the field survey some of these respondents remarked that as long as there are rains, medicinal plants cannot be threatened. This may indicate that rainfall is considered a more important factor in plants sustenance than exploitation/harvesting. Only a small proportion of the respondents (20%) indicated that they were aware that wild medicinal plant populations are declining, while only 4% recognize the fact that some species maybe extinct. On enquiring if the respondents have planted some of the prioritized medicinal plants on their farmlands, 84% had none while 13% had at least two species.

Some of the species that have been planted on farm or deliberately allowed to persist when wild habitats were converted into agricultural lands include: *Croton megalocarpus*, *Aloe secundiflora*, *Azadirachta indica *, *Warburgia ugandensis *, *Ricinus communis *and *Terminalia brownii*. These results are consistent with studies in Peru for example, that have established that although many species are already commercialized, native species are seldom cultivated [30].

Some of the plants whose populations were reported to be decreasing were: *Solanum renschii*, *Populus ilicifolia*, *Strychnos henningsii*, *Rumex usambarensis*. Of these, *Solanum renschii, Populus ilicifolia *were already known to be rare species [40]. Studies involving Kenya on medicinal plants reveal decline of these resources [14]. In other parts of this Province, earlier studies have established that herbalists now have to travel far and wide to collect plant species which were initially common [12]. Consequently, local people in this area may require capacity building and awareness regarding medicinal plants conservation status, domestication strategies as well as appropriate methods of propagation.

## Conclusions

The results of this study reveal the most important medicinal plants of Mwingi district as prioritized by the local people. Some of the plants are already under threat and require conservation measures. Unfortunately, the bulk of the people seem to be unaware of the great threat facing medicinal plants in the wild. The data therefore, presents research, educational and awareness gaps that need to be filled in this area particularly in regard to conservation strategies and sustainable use of medicinal plants.

## Competing interests

The authors declare that they have no competing interests

## Authors' contributions

GNN participated in data collection, data analysis, drafting and submission of the manuscript. IMK participated in data collection, drafting and proof reading of the manuscript. PKN participated in data collection. POO participated in data collection. All authors read and approved the final manuscript.

## Supplementary Material

Additional file 1 **Appendix**. A list of species and voucher specimen numbers Click here for file
