# Supplementary material for: Utilisation of priority traditional medicinal plants and local people's knowledge on their conservation status in arid lands of Kenya (Mwingi District)
Source: J Ethnobiol Ethnomed. 2010 Aug 16;6:22. doi: 10.1186/1746-4269-6-22 (PMC2930631; doi:10.1186/1746-4269-6-22)
Supplement: Additional file 1 — Appendix. A list of species and voucher specimen numbers [file 1746-4269-6-22-S1.DOC]

| species | Voucher specimen number |
| --- | --- |
| *Acacia seyal* Del. | GNN *el al* Mwingi, 26 |
| *Acalypha fruticosa* Forssk. | GNN *el al* Mwingi, 77 |
| *Agave sisalana* Perrine | GNN el al Mwingi, 20 |
| *Ajuga remota* Benth | GNN el al Mwingi, 38 |
| *Albizia amara* (Roxb.) Boiv. | GNN *el al* Mwingi, 15 |
| *Albizia anthelmintica* Brongn. | GNN *el al* Mwingi, 69 |
| *Aloe secundiflora* Engl. | GNN *el al* Mwingi, 30 |
| *Antidesma venosum* Tul | GNN *el al* Mwingi, 25 |
| *Azadirachta indica* A. Juss. | GNN *el al* Mwingi, 90 |
| *Boscia coriacea* Pax | GNN *el al* Mwingi, 70 |
| *Capsicum annuum* L. | GNN *el al* Mwingi, 66 |
| *Capsicum frutescens* L. | GNN *el al* Mwingi, 32 |
| *Carissa edulis* (Forssk.) Vahl | GNN *el al* Mwingi, 78 |
| *Commiphora erythraea* Engl. | GNN *el al* Mwingi, 82 |
| *Croton megalocarpus* Hutch. | GNN *el al* Mwingi, 51 |
| *Ficus sycomorus* L. | GNN *el al* Mwingi, 10 |
| *Hymenodictyon parvifolium* Oliv. | GNN el al Mwingi, 7 |
| *Juniperus procera* Endl. | GNN *el al* Mwingi, 87 |
| *Maytenus senegalensis* (Lam.) Exell | GNN *el al* Mwingi, 61 |
| *Ocimum basilicum* L. | GNN *el al* Mwingi, 43 |
| *Ocimum gratissimum* L | GNN *el al* Mwingi, 56 |
| *Populus ilicifolia* (Engl.) Rouleau | GNN *el al* Mwingi, 29 |
| *Ricinus communis* L. | GNN *el al* Mwingi, 11 |
| *Rumex usambarensis (*Dammer) Dammer | GNN *el al* Mwingi, 6 |
| *Salvadora persica* L. | GNN *el al* Mwingi, 44 |
| *Sclerocarya birrea* (A. Rich.) Hochst. | GNN *el al* Mwingi, 39 |
| *Solanum renschii* Vatke | GNN *el al* Mwingi, 33 |
| *Strychnos henningsii* Gilg | GNN *el al* Mwingi, 85 |
| *Terminalia brownie* Fresen. | GNN *el al* Mwingi, 3 |
| *Vernonia amygdalina* Del. | GNN *el al* Mwingi, 22 |
| *Vigna unguiculata* (L.) Walp. | GNN el al Mwingi, 2 |
| *Warburgia ugandensis s*prague | GNN *el al* Mwingi, 24 |
| *Zanthoxylum chalybeum* Engl. | GNN *el al* Mwingi, 12 |
